# High Transferability of Homoeolog-Specific Markers between Bread Wheat and Newly Synthesized Hexaploid Wheat Lines

**DOI:** 10.1371/journal.pone.0162847

**Published:** 2016-09-09

**Authors:** Deying Zeng, Jiangtao Luo, Zenglin Li, Gang Chen, Lianquan Zhang, Shunzong Ning, Zhongwei Yuan, Youliang Zheng, Ming Hao, Dengcai Liu

**Affiliations:** 1 Triticeae Research Institute, Sichuan Agricultural University at Chengdu, Wenjiang, Sichuan, 611130, China; 2 Crop Research Institute, Sichuan Academy of Agricultural Science, Chengdu, Sichuan, 610066, China; Institute of Genetics and Developmental Biology Chinese Academy of Sciences, CHINA

## Abstract

Bread wheat (*Triticum aestivum*, 2n = 6x = 42, AABBDD) has a complex allohexaploid genome, which makes it difficult to differentiate between the homoeologous sequences and assign them to the chromosome A, B, or D subgenomes. The chromosome-based draft genome sequence of the ‘Chinese Spring’ common wheat cultivar enables the large-scale development of polymerase chain reaction (PCR)-based markers specific for homoeologs. Based on high-confidence ‘Chinese Spring’ genes with known functions, we developed 183 putative homoeolog-specific markers for chromosomes 4B and 7B. These markers were used in PCR assays for the 4B and 7B nullisomes and their euploid synthetic hexaploid wheat (SHW) line that was newly generated from a hybridization between *Triticum turgidum* (AABB) and the wild diploid species *Aegilops tauschii* (DD). Up to 64% of the markers for chromosomes 4B or 7B in the SHW background were confirmed to be homoeolog-specific. Thus, these markers were highly transferable between the ‘Chinese Spring’ bread wheat and SHW lines. Homoeolog-specific markers designed using genes with known functions may be useful for genetic investigations involving homoeologous chromosome tracking and homoeolog expression and interaction analyses.

## Introduction

The number of plant genomes that have been sequenced since the release of the *Arabidopsis thaliana* genome sequence in 2000 continues to grow. Recent genome comparisons have advanced our understanding of the evolution and structure of plant genomes, and the resulting information has been used to improve crop quality and production. Bread wheat (*Triticum aestivum*, 2n = 6x = 42, AABBDD) is a vital crop in terms of food security, providing nearly 20% of the calories and protein consumed by the global population [1]. However, the study of its genetics has been impeded by its complex and large hexaploid genome that contains a high proportion of repetitive DNA [2,3].

The allohexaploid genomic structure of bread wheat was formed by two sequential allopolyploidization events. Additionally, the donors of the wheat subgenomes A, B, and D originated from the same ancestor [4]. The first allopolyploidization event formed the tetraploid wheat *Triticum turgidum* (AABB) through a hybridization between the diploid species *Triticum urartu* (AA) and presumably *Aegilops speltoide*s (SS). Bread wheat was then generated following a second allopolyploidization event 7000–12,000 years ago between *T*. *turgidum* and the wild diploid species *Aegilops tauschii* (DD) [5–7]. By mimicking the origination of bread wheat, artificial allohexaploid wheat lines (i.e., synthetic hexaploid wheat; SHW) can be generated in the laboratory, providing materials containing the genetic diversity of *T*. *turgidum* and *Ae*. *tauschii* that was lost during the origination, domestication, and breeding of modern bread wheat populations [8]. In addition to their applications in wheat breeding programs [9], SHW lines have been widely used for genetic studies to re-examine the biological phenomena and mechanisms that led to the generation of wheat [10]. The highly genetic polymorphism between SHW and bread wheat lines makes SHW lines useful for gene mapping investigations. Moreover, because their genome consists of A, B, and D homoeologous chromosome sets, each of their wheat genes potentially exists as a trio of A, B, and D homoeoloci. This makes newly synthesized allohexaploid wheat with clear hybrid linkages being a desirable model suitable for studying genetic interactions between three homoeologous genomes [11]. However, this genetic structure leads to problems regarding differentiating and assigning the highly conserved homoeologous sequences/markers. Meanwhile, distinguishing homoeologous from homologous markers is complicated and error prone [12–14].

The recent release of the chromosome-based draft genome sequences of the common wheat cultivar ‘Chinese Spring’ and its A and D genome donor species *T*. *urartu* (AA) and *Ae*. *tauschii* (DD) [2,15,16] has enabled the large-scale development of polymerase chain reaction (PCR)-based markers specific for homoeologs from the A, B, and D subgenomes. In this study, we developed chromosome 4B and 7B nullisomic lines that were spontaneously produced from SHW. We designed PCR primers specific for chromosomes 4B and 7B based on the highly differentiated homoeologous genes from the ‘Chinese Spring’ gene models [2]. The results of additional PCR analyses indicated that a high proportion of these markers were specific for chromosomes 4B and 7B of SHW, revealing the high transferability of homoeolog-specific markers between common wheat and SHW.

## Materials and Methods

### Plant materials

We used SHW lines generated from the hybridization between *T*. *turgidum* ssp. *turigidum* line AS313 and *Ae*. *tauschii* ssp. *tauschii* accession AS60. The first SHW generation (S_1_) was obtained following a spontaneous chromosome doubling of triploid F_1_ hybrid plants, which was achieved by the union of unreduced gametes [17]. The S_1_ plants were self-pollinated to produce S_2_, S_3_, and S_4_ generations.

The plant materials were germinated in petri dishes in the normal sowing season for winter wheat in the Sichuan province of China and were then transferred to plastic pots with humus soil and grown under the same outdoor conditions. Before transplanting, root-tips of individual synthesized hexaploid plants were checked for euploidy by multicolor fluorescence in situ hybridization (FISH). The nullisome and euploid lines that were confirmed by cytological observation were subjected to PCR analysis.

### Multicolor fluorescence *in situ* hybridization analysis

Slides were prepared as described by Komuro et al. [18] with minor modifications. The root tips were treated with 1.0 MPa nitrous oxide gas in an air-tight stainless steel chamber for 120 minute and fixed in 100% acetic acid for five minutes. Root tips were immediately washed in distilled water for five minutes. The root meristem was cut and put into a 1.5 ml centrifuge tube containing 10 μL pectolyase Y-23 (1%) (Kikkoman Co., Tokyo, Japan) and cellulase Onozuka R-10 (2%) (Yakult Honsha Co., Ltd. Minato-ku, Tokyo, Japan) in citric buffer (sodium citrate 5 mM, EDTA 5 mM, pH 5.5). The tube was transferred to a 37°C water bath and the root section was digested for 50 minute. After digestion, the root section was washed in distilled water. After aspiration of the distilled water, the root section was washed with 100% ethanol to remove remaining moisture two times. The tube was then agitated several times to disperse the digested cell sections into a suspension of single cells in 20 μL 100% acetic acid.

Clean glass slides were placed on desktop and 10 μL acetic acid cell suspension was dropped onto it. After air dry, the cell spreads on the slides were fixed with a 4% formaldehyde solution for 5 minutes by immersing the slides in a jar. The 4% formaldehyde solution was then washed from the slides by immersing the slides in a jar with 2× SSC.

The multicolor fluorescence *in situ* hybridization (FISH) was completed as described by Tang et al. [19]. Probes Oligo-pSc119.2 and Oligo-pTa535 were 5' end-labelled with 6-carboxytetramethylrhodamine (Tamra) and 6-carboxyfluorescein (6-FAM), respectively. Oligonucleotide probes were synthesized by TSINGKE biological technology company (Chengdu, China). These synthesized probes were diluted in 100 μl 1× TE solution (pH 7.0) as storage fluid and diluted 10 times as working fluid. The probe mixture (0.2 μl working fluid of each probe in 2 × SSC and 1 × TE buffer, pH 7.0, total volume = 10 μl) was dropped at the center of the cell spreads and then covered with glass coverslip. Slides were stored in a moist box at 37°C for 2 h and washed in 2× SSC at room temperature. The slides were mounted with Vectashield mounting medium (Vector Laboratories) with DAPI (4`,6-diamidino-2-phenylindole). An Olympus BX-51 microscope coupled to a Photometric SenSys Olympus DP70 CCD camera (Olympus, Tokyo, Japan) was used to analyze and document the chromosomes.

### Identification of highly differentiated homoeologs and marker design

We compared the high-confidence ‘Chinese Spring’ bread wheat gene models (HC1–HC4) (https://urgi.versailles.inra.fr/download/iwgsc/Gene_models/) [2] with BLASTP (e-value cutoff: 1e-5) to detect genes present only on chromosomes 4B or 7B. The criteria used to identify genes included a minimum of 90% alignment length match and a reciprocal best hit [2,20]. We herein refer to these as highly differentiated homoeologs (HDHs). However, these chromosome-specific genes may have homoeologs with sequence similarities less than 90%.

Although we selected HDHs lacking intact homoeologs on other chromosomes (with sequence similarities > 90%), fragments highly homologous to HDHs were possible because of the relatively low sequence quality of some genes in the current wheat genome database. To design HDH-specific markers more efficiently, randomly selected HDHs were used to search for highly similar ‘Chinese Spring’ cDNA sequences [2] using the BLASTN tool (default settings) of the EnsemblPlants website (http://plants.ensembl.org/Multi/Tools/Blast?db=core#). We downloaded the two most similar sequences from the A or D subgenomes, and aligned them against the target HDH sequences using Primer 5.0 to detect the unmatched sites, including insertion/deletion and single nucleotide polymorphism (SNP) sites. These variations were used to design HDH-specific markers. The 3′ end of the forward primer contained at least two polymorphic sites for all aligned sequences. Polymorphic sites among aligned sequences were unnecessary to design the reverse primer.

### Marker validation

Genomic DNA was isolated from seedling leaves using the Plant Genomic DNA Kit (Tiangen, China). The PCR was completed in 20-μL samples consisting of 8 μL H_2_O, 10 μL 2× Taq PCR MasterMix (Biomed, China), 0.5 μL forward primer, 0.5 μL reverse primer, and 1 μL DNA template (about 100 ng/μL). The PCR program was as follows: 94°C for 5 min; 35 cycles of 95°C for 30 s, 56 to 62°C for 30 s (annealing temperature depended on the primers used), and 72°C for 1 min; 72°C for 10 min. Amplification products were detected by 1.5% agarose gel electrophoresis, and photographed using the Bio-Rad Chemi Doc MP system and Image Lab 4.0 software.

### Construction of genetic maps

The locations of selected genes were determined based on a genetic map from the POPSEQ project by Leibniz Institute of Plant Genetics and Crop Plant Research (IPK) (http://wheat-urgi.versailles.inra.fr/Seq-Repository/Genes-annotations). The genetic map was drawn using the JoinMap 4.0 program.

## Results

### Development of nullisomic lines from synthetic hexaploid wheat

**Fig 1:**
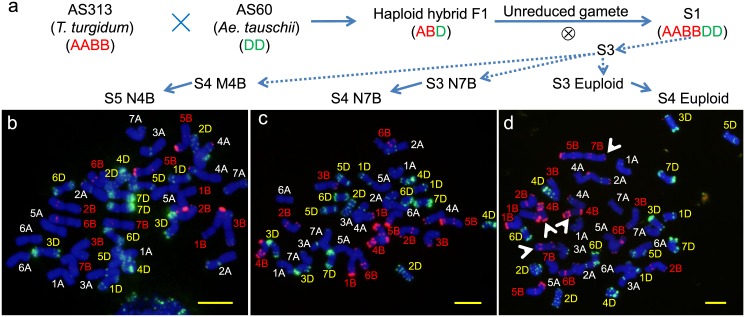
Development of nullisomes from synthetic hexaploid wheat lines and identification by fluorescence *in situ* hybridization. **a** Spontaneous production of euploids and aneuploids from newly synthesized hexaploid wheat. **b** Chromosome 4B nullisome in the S_5_ generation (S5N4B) derived from the 4B monosome in the S_4_ generation (S4M4B). **c** Chromosome 7B nullisome in the S_4_ generation (S4N7B). **d** Euploid. Arrows indicate chromosomes 4B and 7B. Chromosomes were counterstained with DAPI (blue). Oligo-pSc119.2 (red) and Oligo-pTa535 (green) were used as probes. Bar = 10 μm

Randomly selected S_3_ and S_4_ seeds of the SHW line generated from the hybridization between *T*. *turgidum* line AS313 and *Ae*. *tauschii* accession AS60 were used to screen aneuploid plants by multicolor FISH (Fig 1a). The seven analyzed S_3_ seeds corresponded to two euploids and five aneuploids, including two plants (2n = 40 + 1), one plant (2n = 39 + 1), one plant (2n = 39), and one 7B nullisome. The chromosomal fragments were derived from the B genome. Out of the analyzed nine S_4_ seeds, six were euploids and three were aneuploids, including one 4B monosome, one 4B trisome, and one plant (2n = 41) with an unclear chromosomal constitution. These results indicated the analyzed populations exhibited a high degree of chromosomal variability.

The seeds harvested from the 4B monosome, 7B nullisome, and one of the S_3_ euploids were used to screen for additional nullisomes and euploids (Fig 1a). Out of the 33 individuals from the 4B monosome, four were for true 4B nullisomes that lacked a pair of 4B chromosomes. They contained the other 20 pairs of wheat chromosomes, and did not exhibit any obvious chromosome structural changes (Fig 1b). Meanwhile, we identified 13 true 7B nullisomes (Fig 1c). Out of 28 analyzed S_4_ seeds produced from the S_3_ euploid (Fig 1d), 23 were for true euploids, while the other five corresponded to aneuploids. These results indicated that the true euploids produced new aneuploids.

### Primer design based on ‘Chinese Spring’ gene models

We compared the HC1–HC4 ‘Chinese Spring’ gene models. Out of the 4,467 analyzed genes on chromosome 4B and 4,015 genes on chromosome 7B, 2,057 (46.0%) on 4B (814 on 4BS and 1,243 on 4BL) and 2,202 (54.8%) on 7B (795 on 7BS and 1,407 on 7BL) had no homoeologous genes on the other chromosomes. Homoeologous genes were considered those with a minimum of 90% sequence length similarity. The number of HDHs may have been overestimated because of the relatively poor quality of the gene models for the current wheat genome, which was assembled using short reads from separate chromosome shotgun sequencing experiments.

**Fig 2:**
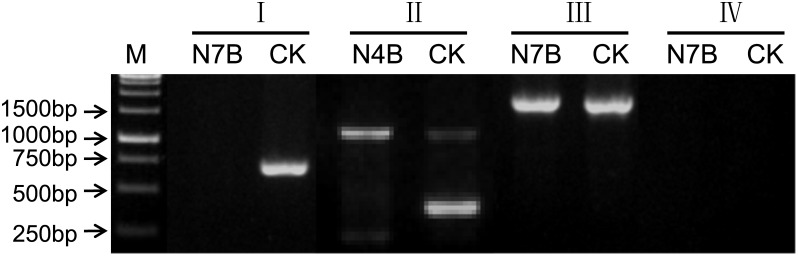
Example of a homoeolog-specific marker designed based on a homoeologous gene alignment of partial sequences. Green arrows indicate the locations of the forward and reverse primers. Red arrows indicate the three SNP sites.

We used 93 and 90 randomly selected homoeologs from chromosomes 4B and 7B, respectively, to design homoeolog-specific markers. Based on alignments of highly similar sequences, regions containing at least two polymorphic sites were used to design the forward primer specific for the 3′ end. A primer based on three SNP sites is presented in Fig 2. Detailed information regarding 183 primer pairs is provided in S1 Table.

### Homoeolog-specific markers confirmed by PCR amplification

**Fig 3:**
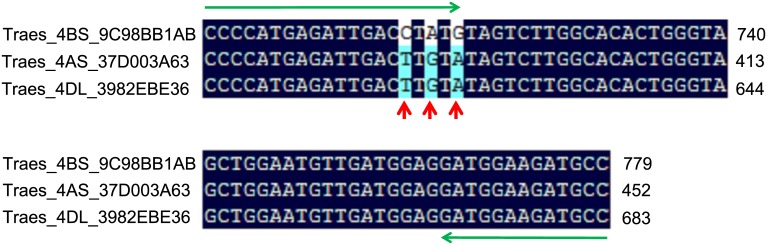
Examples of four types of PCR amplification patterns for the chromosome 7B (N7B) and 4B (N4B) nullisomes and a euploid (CK) line. The four genes used to design primers were *Traes_7BS_AC4692F05* (Type I), *Traes_4BS_EB4728825* (Type II), *Traes_7BS_F9BB82D98* (Type III), and *Traes_7BS_76FF7AB3F* (Type IV).

To test the efficiency of homoeolog-specific markers designed for ‘Chinese Spring’ gene sequences in the SHW genetic background, 183 primer pairs were used in PCR amplifications. Based on the amplification patterns observed for the euploids and nullisomes, the PCR-based markers were classified as one of the following four types (Fig 3; Table 1):

1. Markers that produced polymorphic amplification patterns with specific amplification of the target chromosome (Fig 3I). Nullisomes lacked an amplification product, while disomes produced a distinct target band. A total of 55 (59.1%) and 60 (66.7%) markers for chromosomes 4B and 7B, respectively, belonged to this marker type.
2. Markers that produced polymorphic amplification patterns with specific and non-specific amplification products (Fig 3II). In addition to the target amplicon, which only appeared in the disomic line, extra amplification products were produced for nullisomes and disomes. This category consisted of only two markers for chromosome 4B. The sequence corresponding to the missing amplicon in nullisomes was homoeolog-specific.
3. Markers that produced monomorphic amplification patterns (Fig 3III). Nullisomes and disomes generated amplification products that were the same size, including 19 (20.4%) and 20 (22.2%) markers for chromosomes 4B and 7B, respectively. These markers were not homoeolog-specific.
4. Markers that did not generate amplification products (Fig 3IV). Amplicons were not detected for nullisomes and disomes, including 17 (18.3%) and 12 (11.1%) markers for chromosomes 4B and 7B, respectively. Because the primers were designed according to the ‘Chinese Spring’ genome sequence, the lack of amplification products may have reflected the genetic variability between ‘Chinese Spring’ and SHW lines.

**Table 1:** Chromosomal distribution of different marker types.

Chromosome	I	II	III	IV	Total
4B	55	2	19	17	93
7B	60	0	20	10	90

**Fig 4:**
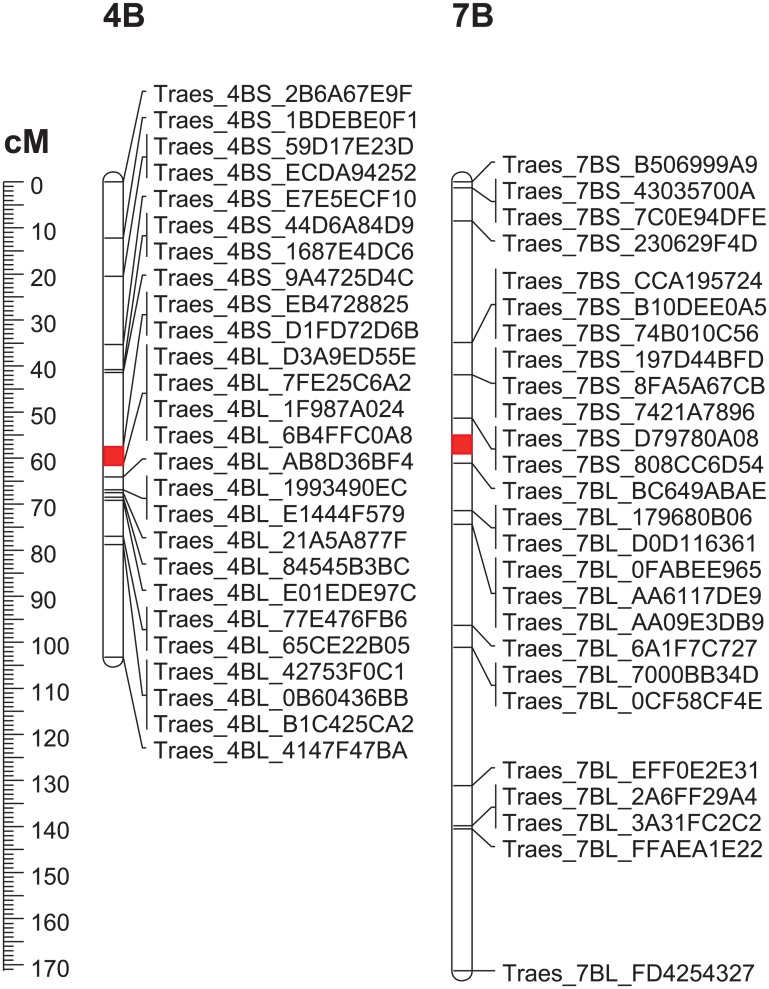
Distribution of homoeolog-specific markers on chromosomes 4B and 7B.

In total, 117 of 183 markers (63.9%; Types I and II; 57 for 4B and 60 for 7B) were suitable homoeolog-specific markers in the synthetic wheat background (S1 Table). Based on the genetic map from the POPSEQ project, we anchored 26 of 57 and 26 of 60 markers on chromosomes 4B and 7B, respectively (Fig 4). The locations of the other markers were unknown because their genes had not been mapped.

## Discussion

Synthetic hexaploid wheat lines represent important materials for wheat genetics research and breeding programs. Because very few ancestor lines of *T*. *turgidum* and *Ae*. *tauschii* were involved in the generation of bread wheat, a considerable amount of genetic variation in the two ancestor species is missing from modern bread wheat populations. The genetic diversity of bread wheat was further limited by domestication and modern breeding processes. The SHW lines enable the simultaneous re-use of *T*. *turgidum* and *Ae*. *tauschii*, and thus have been preferentially used by several research groups to enhance the genetic diversity of bread wheat [8,9,21,22]. Because they have clear hybrid and parental linkages, SHW lines are also ideal genetic materials for evolutionary biology studies [10].

Aneuploidy is a common phenomenon in new SHW lines. In this study, we observed a high frequency of chromosomal variation in progenies of new SHW lines generated from hybridizations between *T*. *turgidum* AS313 and *Ae*. *tauschii* AS60. Aneuploid plants are produced from triploid F_1_ hybrids or hexaploid plants. The SHW aneuploids may be spontaneously generated by the union of aneuhaploid gametes, which are derived from meiotic restitution of the F_1_ hybrids resulting from the cross between *T*. *turgidum* and *Ae*. *tauschii*, or because of cytological instability of new SHW lines. The formation of unreduced gametes is normal in the triploid F_1_ hybrids of a cross between *T*. *turgidum* and *Ae*. *tauschii*, and their union will result in the spontaneous production of hexaploid wheat S_1_ seeds [17]. Although most of the generated S_1_ seeds will be euhexaploid (2n = 42), aneuploids (2n = 40, 41, 43, or 44) will also be frequently observed because of the existence of aneuhaploid gametes (n = 20 or 22) [23]. The aneuploid frequency in S_1_ plants depends on the hybrid combinations. In a recent study, the frequency of aneuploids in the AS313 × AS60 cross (33.3%) was higher than that of the LDN × AS60 cross (13.2%). This observation is related to the differences in the ability to form unreduced gametes [24]. A high proportion of aneuploid plants can also be produced in the selfing progenies of euploid SHW lines as shown in this study and previous reports [25,26]. However, the underlying genetic mechanisms are still unknown. Although we only identified the aneuploid plants related to chromosomes 4B and 7B, we expect that aneuploid plants involving all 21 wheat chromosomes derived from bread wheat ancestor species will eventually be generated for the development of molecular markers specific for individual chromosomes.

The chromosome-based draft genome sequence of the ‘Chinese Spring’ common wheat cultivar is very useful for developing homoeolog-specific markers in the SHW background. In this study, 183 putative homoeolog-specific markers were designed according to the ‘Chinese Spring’ genome sequence. Up to 64% of the markers were homoeolog-specific in the SHW background, which indicates that homoeolog-specific markers are highly transferable between ‘Chinese Spring’ bread wheat and SHW lines. Although the bread wheat and SHW line were derived from an interspecific hybridization between *T*. *turgidum* and *Ae*. *tauschii*, the SHW line was new, while the bread wheat cultivar originated a long time ago [4]. The high transferability indicates homologous genes are highly conserved between bread wheat and its donor species. This is consistent with the fact that gene sequences are highly conserved between the ‘Chinese Spring’ subgenomes and their respective diploid relatives [2,4]. However, about 36% of the markers that were assumed to be specific for individual ‘Chinese Spring’ chromosomes were either not homoeolog-specific or failed to produce PCR products in the SHW background. There are potential explanations for these results. For example, we did not confirm the presence of the markers in the ‘Chinese Spring’ background. Some markers may have been incorrectly considered homoeolog-specific because of the relatively low quality of the gene models in the current wheat genome release. Genetic variation between ‘Chinese Spring’ and SHW lines may also be responsible for our observations.

The homoeolog-specific markers can be used for chromosome tracking. In new amphidiploid lines produced from wheat and alien species, chromosomal variations are common because of cytological instability [8]. Among the analyzed SHW plants, we detected both aneuploids as well as plants with fragmented chromosomes. Cytological techniques, such as sequential multi-color genomic *in situ* hybridization and FISH, have been widely used to identify chromosomal constituents. However, they are highly technical procedures that are labor-intensive, time consuming, and costly. As indicated by the identification of chromosome 4B and 7B nullisomes in this study, homoeolog-specific markers based on PCR amplification represent simple and highly efficient tools to detect nullisomes. Homoeolog-specific markers can also be used to identify missing wheat chromosomal fragments. Wheat—alien substitution and translocation lines are important materials for transferring desirable genes from alien wheat species by distant hybridization. In a substitution line, a pair of wheat chromosomes are replaced by a pair of alien chromosomes. In contrast, in a translocation line, wheat chromosomal fragments are replaced by alien chromosomal segments. Homoeolog-specific markers may be helpful for developing substitution and translocation lines.

## Supporting Information

S1 Table Details of the 183 primers for chromosomes 4B and 7B designed in this study. (XLSX)

## References

[1] Hawkesford MJ, Araus JL, Park R, Calderini D, Miralles D, Shen T, et al Prospects of doublingglobal wheat yields. Food & Energy Security. 2013; 2: 34–48.

[2] IWGSC. A chromosome-based draft sequence of the hexaploid bread wheat (*Triticum aestivum*) genome. Science. 2014; 345: 1251788 10.1126/science.1251788 25035500

[3] Wang Y, Drader T, Tiwari VK, Dong L, Kumar A, Huo N, et al Development of a D genome specific marker resource for diploid and hexaploid wheat. Bmc Genomics. 2015; 16: 646 10.1186/s12864-015-1852-2 26315263 PMC4552153

[4] Marcussen T, Sandve SR, Heier L, Spannagl M, Pfeifer M, Jakobsen KS, et al Ancient hybridizations among the ancestral genomes of bread wheat. Science. 2014; 345: 1250092 10.1126/science.1250092 25035499

[5] Huang S, Sirikhachornkit A, Su X, Faris J, Gill B, Haselkorn R, et al Genes encoding plastid acetyl-CoA carboxylase and 3-phosphoglycerate kinase of the *Triticum*/*Aegilops* complex and the evolutionary history of polyploid wheat. Proceedings of the National Academy of Sciences, USA. 2002; 99: 8133–8138. 10.1073/pnas.072223799 PMC123033 12060759

[6] Kihara H. Discovery of the DD-analyser, one of the ancestors of *Triticum vulgare* (abstr) (in Japanese). Agriculture and Horticulture. 1944; 19: 889–890.

[7] McFadden ES, Sears ER. The artificial synthesis of *Triticum spelta*. Records of the Genetic Society of America. 1944; 13: 26–27.

[8] Liu D, Hao M, Li A, Zhang L, Zheng Y, Mao L (2016) Allopolyploidy and Interspecific Hybridization for Wheat Improvement In: Mason AS, editor. Polyploidy and hybridization for crop improvement: CRC Press pp. 27–52.

[9] Yang W, Liu D, Li J, Zhang L, Wei H, Hu X, et al Synthetic hexaploid wheat and its utilization for wheat genetic improvement in China. Journal of Genetics and Genomics. 2009; 36: 539–546. 10.1016/S1673-8527(08)60145-9 19782955

[10] Li AL, Geng SF, Zhang LQ, Liu DC, Mao L. Making the bread: insights from newly synthesized allohexaploid wheat. Molecular Plant. 2015; 8: 847–859. 10.1016/j.molp.2015.02.016 25747845

[11] Li A, Liu D, Liu D, Wu J, Zhao X, Hao M, et al mRNA and Small RNA transcriptomes reveal insights into dynamic homoeolog regulation of allopolyploid heterosis in nascent hexaploid wheat. The Plant Cell. 2014; 26: 1878–1900. 24838975 10.1105/tpc.114.124388 PMC4079356

[12] Poole R, Barker G, Wilson ID, Coghill JA, Edwards KJ. Measuring global gene expression in polyploidy; a cautionary note from allohexaploid wheat. Functional & Integrative Genomics. 2007; 7: 207–219. 17364174 10.1007/s10142-007-0046-7

[13] Barker GLA, Edwards KJ. A genome-wide analysis of single nucleotide polymorphism diversity in the world's major cereal crops. Plant Biotechnology Journal. 2009; 7: 318–325. 10.1111/j.1467-7652.2009.00412.x 19386040

[14] Allen AM, Barker GLA, Berry ST, Coghill JA, Gwilliam R, Kirby S, et al Transcript-specific, single-nucleotide polymorphism discovery and linkage analysis in hexaploid bread wheat (*Triticum aestivum* L.). Plant Biotechnology Journal. 2011; 9: 1086–1099. 10.1111/j.1467-7652.2011.00628.x 21627760

[15] Jia J, Zhao S, Kong X, Li Y, Zhao G, He W, et al *Aegilops tauschii* draft genome sequence reveals a gene repertoire for wheat adaptation. Nature. 2013; 496: 91–95. 10.1038/nature12028 23535592

[16] Ling HQ, Zhao S, Liu D, Wang J, Sun H, Zhang C, et al Draft genome of the wheat A-genome progenitor *Triticum urartu*. Nature. 2013; 496: 87–90. 23535596 10.1038/nature11997

[17] Zhang LQ, Liu DC, Zheng YL, Yan ZH, Dai SF, Li YF, et al Frequent occurrence of unreduced gametes in *Triticum turgidum*–*Aegilops tauschii hybrids*. Euphytica. 2010; 172: 285–294.

[18] Komuro S, Endo R, Shikata K, Kato A. Genomic and chromosomal distribution patterns of various repeated DNA sequences in wheat revealed by a fluorescence in situ hybridization procedure. Genome. 2013; 56: 131–137. 10.1139/gen-2013-0003 23659696

[19] Tang Z, Yang Z, Fu S. Oligonucleotides replacing the roles of repetitive sequences pAs1, pSc119.2, pTa-535, pTa71, CCS1, and pAWRC.1 for FISH analysis. Journal of Applied Genetics. 2014; 55: 313–318. 10.1007/s13353-014-0215-z 24782110

[20] Tatusov RL, Koonin EV, Lipman DJ. A genomic perspective on protein families. Science. 1997; 278: 631–637. 9381173 10.1126/science.278.5338.631

[21] Mujeeb-Kazi A, Rosas V, Roldan S. Conservation of the genetic variation of *Triticum tauschii* (Coss.) Schmalh.(*Aegilops squarrosa* auct. non L.) in synthetic hexaploid wheats (*T*. *turgidum* L. s. lat. x *T*. *tauschii*; 2n = 6x = 42, AABBDD) and its potential utilization for wheat improvement. Genetic Resources and Crop Evolution. 1996; 43: 129–134.

[22] Ogbonnaya FC, Abdalla O, Mujeeb-Kazi A, Kazi AG, Xu SS, Gosman N, et al Synthetic hexaploids: harnessing species of the primary gene pool for wheat improvement. Plant Breeding Reviews. 2013; 37: 35–122.

[23] Zhang L, Chen Q, Yuan Z, Xiang Z, Zheng Y, Liu D. Production of aneuhaploid and euhaploid sporocytes by meiotic restitution in fertile hybrids between durum wheat Langdon chromosome substitution lines and *Aegilops tauschii*. Journal of Genetics & Genomics. 2008; 35: 617–623. 18937918 10.1016/S1673-8527(08)60082-X

[24] Hao M, Luo J, Zeng D, Zhang L, Ning S, Yuan Z, et al *QTug*.*sau-3B* is a major quantitative trait locus for wheat hexaploidization. G3-Genes Genomes Genetics. 2014; 4: 1943–1953. 10.1534/g3.114.013078 PMC4199700 25128436

[25] Mestiri I, Chague V, Tanguy AM, Huneau C, Huteau V, Belcram H, et al Newly synthesized wheat allohexaploids display progenitor-dependent meiotic stability and aneuploidy but structural genomic additivity. New Phytologist. 2010; 186: 86–101. 10.1111/j.1469-8137.2010.03186.x 20149116

[26] Zhang H, Bian Y, Gou X, Zhu B, Xu C, Qi B, et al Persistent whole-chromosome aneuploidy is generally associated with nascent allohexaploid wheat. Proceedings of the National Academy of Sciences, USA. 2013; 110: 3447–3452. 10.1073/pnas.1300153110 PMC3587266 23401544

